# Zika Virus Outbreak, Barbados, 2015–2016

**DOI:** 10.4269/ajtmh.17-0978

**Published:** 2018-04-09

**Authors:** Sadie J. Ryan, Catherine A. Lippi, Colin J. Carlson, Anna M. Stewart-Ibarra, Mercy J. Borbor-Cordova, Moory Romero, Shelly-Ann Cox, Roché Mahon, Adrian Trotman, Leslie Rollock, Marquita Gittens-St. Hilaire, Desmond King, Steven Daniel

**Affiliations:** 1 Quantitative Disease Ecology and Conservation Lab, Department of Geography, University of Florida, Gainesville, Florida;; 2 Emerging Pathogens Institute, University of Florida, Gainesville, Florida;; 3 School of Life Sciences, University of KwaZulu-Natal, Durban, South Africa;; 4 National Socio-Environmental Synthesis Center, University of Maryland, Annapolis, Maryland;; 5 Department of Biology, Georgetown University, Washington, DC;; 6 Center for Global Health and Translational Science, State University of New York (SUNY) Upstate Medical University, Syracuse, New York;; 7 Facultad de Ingeniería Marítima, Ciencias Oceánicas y Recursos Naturales (FIMCBOR), Escuela Superior Politécnica del Litoral (ESPOL), Guayaquil, Ecuador;; 8 Caribbean Institute for Meteorology and Hydrology (CIMH), Bridgetown, Barbados;; 9 Ministry of Health, St. Michael, Barbados;; 10 Faculty of Medical Sciences, University of the West Indies at Cave Hill, Bridgetown, St. Michael, Barbados;; 11 Barbados Leptospira Laboratory, Ministry of Health, St. Michael, Barbados

## Abstract

Barbados is a Caribbean island country of approximately 285,000 people, with a thriving tourism industry. In 2015, Zika spread rapidly throughout the Americas, and its proliferation through the Caribbean islands followed suit. Barbados reported its first confirmed autochthonous Zika transmission to the Pan American Health Organization in January 2016, a month before the global public health emergency was declared. After detection of suspected Zika cases on Barbados in 2015, 926 individuals were described as suspected cases, and 147 laboratory-confirmed cases were reported through December 2016, the end of the most recent epidemiological year. In this short report, we describe the epidemiological characteristics of 926 clinical case records that were originally suspected as cases of Zika, and which were subsequently sent for testing and confirmation; 147 were found positive for Zika, using reverse transcription-polymerase chain reaction methods, another 276 tested negative, and the remaining 503 were either pending results or still in the suspected category. Women were represented at about twice the rate of men in case records where gender was reported (71.9%), and confirmed cases (78.2%), and 19 of the confirmed positive cases were children under the age of 10.

Zika virus (ZIKV), a flavivirus transmitted primarily by *Aedes aegypti* and *Ae. albopictus* mosquitoes, was first reported outside of Africa and Asia in 2007. However, it was not until 2015 that ZIKV rapidly spread from Brazil throughout the Americas. Initially, regarded as a mild febrile illness, the emergence of associated health complications, such as ZIKV congenital syndrome, including microcephaly and other birth defects, and ZIKV-associated Guillain–Barré syndrome, has posed an unprecedented challenge to global health.^1,2^ Echoing the rapid spread throughout mainland South America, ZIKV reached the Caribbean early in the pandemic. Autochthonous transmission in Martinique was first reported in the epidemiological week (EW) 51 of 2015, the first case from Puerto Rico was reported in the EW 52 of 2015,^3^ and many other islands began reporting cases early in 2016.^4^ However, case data from several countries have yet to be consolidated and described outside of reports by the Pan American Health Organization (PAHO).

Suspected clinical ZIKV disease cases in Barbados were defined using clinical guidelines provided by PAHO, which include a rash plus one or more of the following: fever ≥ 38.5°C, conjunctivitis, arthralgia, myalgia, and periarticular edema,^5^ but laboratory testing of suspected arboviral cases was also conducted during the Barbados ZIKV outbreak. The active surveillance of ZIKV cases (suspected and confirmed) among persons who visited health clinics started as early as May 2015, and the first laboratory-confirmed autochthonous case of ZIKV was reported to PAHO in the EW 1 of 2016. However, there were three cases from December, 2015, which were later laboratory confirmed, of which only one had a travel history during the month of infection. Therefore, asymptomatic cases may have existed before December 2015. Initial ZIKV case confirmation was conducted using the Centers for Disease Control (CDC) Trioplex RT-PCR assay for dengue virus (DENV), Chikungunya virus (CHIKV), and ZIKV, at the Caribbean Public Health Agency laboratory in Trinidad and Tobago, until RT-PCR using the CDC Trioplex assay was established in September 2016 at the Leptospira Laboratory, the national reference laboratory of the Ministry of Health of Barbados. Initial testing was biased toward women, particularly pregnant women, reflecting a targeted response. Once testing capability and capacity became local, all samples with suspected arboviral infection were tested for ZIKV, CHIKV, and DENV. We collected data from records at the Ministry of Health, Barbados, on patients’ age, gender, date of illness onset, occupation, and laboratory diagnostic status (suspected, negative, positive, and pending testing). Our reported total suspected case records comprise clinically suspected ZIKV cases before September of 2016 and all cases tested as suspected for any of the three arboviral infections, after local testing capacity was established.

The first confirmed ZIKV case in Barbados, a 42-year-old man, reported onset on December 26, 2015, during the EW 51. New cases were subsequently recorded through December 30, 2016 (Figure 1), with the last cases in 2016 recorded in the EW 52. In total, 926 cases with ZIKV status (suspected, negative, positive, and pending testing) were recorded in Barbados in 2015 and 2016, after the first confirmed ZIKV case, of which 147 (15.9%) were positively confirmed with RT-PCR and 276 tested negative. The remaining cases were in the suspected and pending status at the time of this analysis.

**Figure 1.**
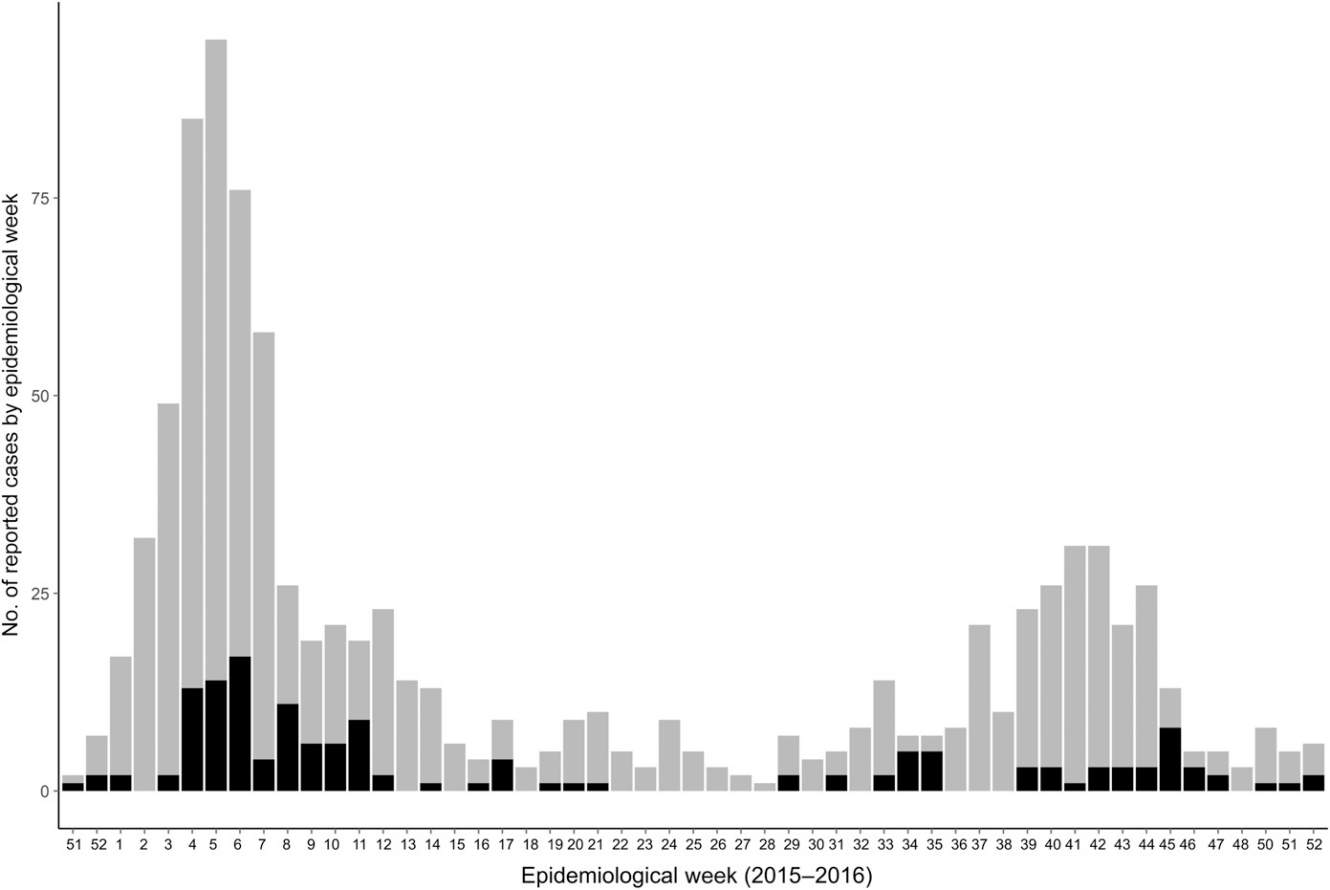
ZIKV cases (suspected in grey, confirmed in black) reported on Barbados (2015–2016).

Gender was reported for 899 of the 926 overall cases in this study (97.1%). Women were disproportionately represented at more than twice the frequency of men, with 646 women (71.9%) and 253 men (28.1%). In the confirmed positive cases, this also bore out with 115 women (78.2%) and 32 men (21.8%) in the positively confirmed cases, consistent with a female bias found in other reports on ZIKV outbreaks.^6^ Age was reported for 875 of 926 overall cases (94.5%), with a mean age of 33 and median age of 32; in the positively confirmed cases, mean age was 30 and median of 31, a difference that was not significant. Of the 926 overall cases, 147 (17.0%) were children under the age of 10 and 573 (65.4%) were of childbearing age (15–49 years). Women of childbearing age represented 77 (52.3%) of the 147 confirmed positive cases and made up a sizable proportion (48.6%) of the 875 overall cases for which age and gender were both reported. For the 147 positive cases in which age was reported, 19 were children under the age of 10. One of 116 unique occupations were reported for 283 records. We grouped these into five major categories: educational (53), service/hospitality (88), health sector (17), administrative/professional (93), and others (32). The most numerous unique occupational descriptions among these were students (39 plus 2 student nurses), teachers (13), nurses (10), and unemployed (15). The high number of unique occupational descriptions reported and the low sample of recorded occupations precludes rigorous statistical inference of occupational hazards. The testing status for other arboviral infections for the 926 clinical cases examined for ZIKV are given in Table 1. It is important to note that DENV testing is conducted at the local reference laboratory of the Ministry of Health, in which blood samples from suspected DENV cases are sent to the laboratory from the clinic and tested for non-structural protein 1 (NS1), Immunoglobulin M (IgM), and Immunoglobulin G (IgG); if the sample is from the first 5 days of illness and if NS1 and IgM are negative within the first 3 days, another test is conducted for IgM and IgG after 5 days of illness.^7^ In these suspected ZIKV case records, we cannot distinguish between the trioplex results and blood test results as part of normal surveillance for DENV. We therefore have far more information about the DENV status than for CHIKV. Of the 926 cases in this study, 314 were positive for DENV, and three for CHIKV, with an additional 75 suspected for CHIKV. Interestingly, there were 15 positively confirmed ZIKV/DENV coinfections, but none of the three reported confirmed CHIKV cases were coinfections. Other factors of interest when reporting ZIKV, such as pregnancy status and access to medical care, were not included in the available data for this report.

**Table 1:** Summary of arboviral infection status (DENV, ZIKV, CHIKV) reported in the 926 total case records

	DENV	ZIKV	CHIKV
Positive	314*	147	3
Negative	601	276	477
Suspected	0	454	75
Pending	0	49	0
Missing status	11	0	371

* DENV status includes blood test results; there were 15 DENV/ZIKV coinfected cases.

*Aedes* mosquitoes are established throughout the Caribbean, with active transmission of DENV, CHIKV, and now ZIKV documented on many islands. In the broader context of emerging arboviruses, the early and rapid onset of the ZIKV outbreak in Barbados relative to the larger pandemic in the Americas demonstrates that the existence of *Aedes* populations leave even the small islands highly susceptible to the spread of novel pathogens. We saw a female bias in cases, particularly toward women of childbearing age, and what appeared to be two waves of cases in 2016 (Figure 1). The rapid proliferation of ZIKV infections calls attention to the need to strengthen local capacities for targeted vector control, integrated strategies such as campaigns for cleaning reservoirs, particularly underground cisterns, and health education through formal and informal education programs. In addition, this calls for global efforts to support the development of effective vaccines and a better understanding of the role of sexual transmission and heightened risk to vulnerable populations such as pregnant women.
